# Pathogenic variants of the mitochondrial aspartate/glutamate carrier causing citrin deficiency

**DOI:** 10.1016/j.tem.2022.05.002

**Published:** 2022-06-17

**Authors:** Sotiria Tavoulari, Denis Lacabanne, Chancievan Thangaratnarajah, Edmund R.S. Kunji

**Affiliations:** 1 Medical Research Council Mitochondrial Biology Unit, University of Cambridge, The Keith Peters Building, Cambridge Biomedical Campus, Hills Road, Cambridge CB2 0XY, UK

## Abstract

Citrin deficiency is a pan-ethnic and highly prevalent mitochondrial disease with three different stages: neonatal intrahepatic cholestasis (NICCD), a relatively mild adaptation stage, and type II citrullinemia in adulthood (CTLN2). The cause is the absence or dysfunction of the calcium-regulated mitochondrial aspartate/glutamate carrier 2 (AGC2/SLC25A13), also called citrin, which imports glutamate into the mitochondrial matrix and exports aspartate to the cytosol. In citrin deficiency, these missing transport steps lead to impairment of the malate-aspartate shuttle, gluconeogenesis, amino acid homeostasis, and the urea cycle. In this review, we describe the geological spread and occurrence of citrin deficiency, the metabolic consequences and use our current knowledge of the structure to predict the impact of the known pathogenic mutations on the calcium-regulatory and transport mechanism of citrin.

## Citrin deficiency is a highly prevalent and complex mitochondrial disease

Citrin deficiency has two major, age-related clinical manifestations: NICCD, presenting in the first year of life, and adult-onset CTLN2 [1,2]. NICCD is characterized by jaundice, failure to thrive, hypoproteinemia, hypoglycemia, multiple aminoacidemias including citrullinemia, and a fatty liver [1,3]. After patients recover from NICCD, they go through an adaptation stage characterized by strong food preferences and a variety of milder clinical symptoms [3]. However, some patients at this stage suffer from failure to thrive and dyslipidemia (FTTDCD) [4]. In adult life, a subset of patients develop CTLN2, the most severe form, which can lead to premature death and is characterized by frequent attacks of hyperammonemia, liver steatosis, neuropsychiatric symptoms, and brain edema [5]. The striking range of symptoms and levels of severity observed within each stage have been reviewed elsewhere [1,3,6–8], but are not well understood.

Citrin deficiency was first described six decades ago [9] and was originally characterized as a rare disease, localized to Japan and East Asia. The incidence in Japan has been estimated to be 1:17 000 with a disease-associated allele occurrence varying between 1:65 in the South [10] and 1:42 in the North [11]. Recent reports in China have shown that the occurrence is 1:45, reaching levels of 1:28 in Southern China [12]. More recently, it has become clear that citrin deficiency is a pan-ethnic disease, and increasing numbers of patients of non-Asian origin are being diagnosed worldwide [13–19].

Citrin deficiency is inherited in an autosomal recessive manner and is caused by pathogenic variants of the *SLC25A13* gene, which encodes the mitochondrial aspartate/glutamate carrier isoform 2 (AGC2), also called citrin [20,21]. Citrin is located in themitochondrial innermembrane and is responsible for the symport of glutamate with a proton into the mitochondrial matrix and the export of aspartate from the matrix to the cytoplasm [21–26], a critical step in the urea cycle [27], gluconeogenesis [28], the malate-aspartate shuttle [29,30], and metabolic energy production.

There are two human isoforms of the mitochondrial aspartate/glutamate carrier. The *SLC25A13* gene product citrin (AGC2) is predominantly expressed in non-excitable tissues such as the liver, kidney, heart, and pancreas, whereas the *SLC25A12* gene product, also known as aralar or AGC1 [31], is predominantly expressed in excitable tissues such as the brain, heart, and skeletal muscle. Recent proteomic studies, however, have shown that both citrin and aralar are expressed in the liver at a ratio of 13:1 and in the brain at a ratio of 1:5 [32]. Nevertheless, owing to different tissue distributions, AGC2 mutations cause citrin deficiency, affecting primarily the liver, whereas AGC1 mutations lead to AGC1 deficiency and defective myelin synthesis, associated with hypotonia, arrested psychomotor development, seizures, spasticity, epilepsy, hypomyelination, and cerebral atrophy [33,34].

Structural studies from our laboratory have shed light on the unique three-domain structure of the aspartate/glutamate carriers [35–37] which comprise a calcium-regulated N-terminal domain [38], a mitochondrial carrier domain responsible for substrate transport, and a C-terminal domain with an **amphipathic helix** (see Glossary) that is potentially involved in calcium regulation [35]. They exist as structural homodimers through association of the N-terminal domains, whereas the carrier domains do not interact [35].

Despite major efforts in the past two decades to diagnose, understand, and treat citrin deficiency, major aspects of the underlying molecular and cellular mechanisms remain unknown. In this review, we discuss the metabolic consequences of citrin dysfunction in relation to clinical symptoms and summarize the plethora of pathogenic variants which are placed in a structural, mechanistic, and bioenergetic context by recent advances in the structural mechanism of transport [36,37] and calcium regulation [35].

## Impact of citrin deficiency on metabolism

**Figure 1:**
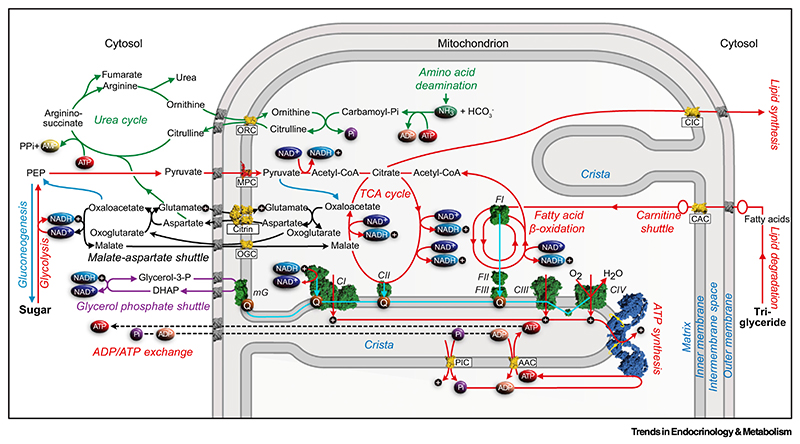
The role of citrin in metabolism. Transport steps and biochemical pathways involved in glycolysis/tricarboxylic acid (TCA) cycle (red), respiratory chain (cyan), gluconeogenesis (blue), malate-aspartate shuttle (black), glycerol phosphate shuttle (purple), and ammonia fixation/urea cycle (green) are shown schematically. Citrin is shown in yellow together with the other mitochondrial carriers: ADP/ATP carriers (AAC), carnitine/acylcarnitine carrier (CAC), citrate carrier (CIC), oxoglutarate carrier (OGC), ornithine carrier (ORC), phosphate carrier (PIC). The respiratory chain complexes 1 to 4 (CI–CIV), mitochondrial glycerophosphate dehydrogenase (mGPD) and acyl-CoA dehydrogenases (FI), electron transfer flavoprotein (FII), and ETF-ubiquinone oxidoreductase (FIII) are shown in green, the dimer of ATP synthase in blue, and the mitochondrial pyruvate carrier heterodimer (MPC) in red/orange. The voltage-gated anion channel (VDAC) in the outer membrane is shown in gray. Key metabolites such as phosphate (Pi, purple), ADP (orange), ATP (red), NAD^+^ (dark blue), NADH (light blue), ammonia (green), and ubiquinone (Q, brown) are shown, and protons as black circles with a plus sign.

Citrin deficiency could impact on many different interlinked metabolic pathways in the cell (Figure 1), leading to the various metabolic phenotypes observed in patients. However, the complete metabolic impact of citrin deficiency has not been fully elucidated due to lack of appropriate model systems. In this section, we attempt to rationalize and discuss the metabolic pathways likely to be affected in citrin deficiency, with a focus on their possible impact on related pathological symptoms.

One of the major metabolic pathways affected by the absence or dysfunction of citrin is the malate-aspartate shuttle [30]. NADH, produced by glycolysis in the cytosol, needs to be converted to NAD^+^, as otherwise glycolysis and other pathways are halted by allosteric modulation. The major pathway for this to be achieved is the malate-aspartate shuttle [29,30], which via a series of enzymatic and transport steps oxidizes NADH in the cytosol and reduces NAD^+^ in the mitochondrial matrix (Figure 1). Citrin together with the mitochondrial oxoglutarate carrier (OGC) [29,39] are crucial for the function of the malate-aspartate shuttle in liver, as for instance shown in mice [40,41]. Although not directly demonstrated in citrin-deficiency patients, it has been proposed that an impaired shuttle leads to an increase in the cytosolic NADH:NAD^+^ ratio [42]. As a consequence, hepatic glycolysis is impaired and fewer electrons enter the respiratory chain at the level of complex I from mitochondrial NADH, leading to a reduction in ATP production from carbohydrates. The alternative pathway for NADH oxidation is the glycerol phosphate shuttle (Figure 1), but in human liver, this is active at a lower level than in brown adipose tissue, skeletal muscle, and brain [43]. This is likely to be the reason why citrin-deficiency patients prefer to eat a high-fat and -protein diet rather than carbohydrates [44,45] as metabolic energy generation from fat does not require a functional malate-aspartate shuttle.

Citrin deficiency may also cause dyslipidemia in the adaptation stage and fatty liver disease in all stages [3]. It is currently not known whether these defects can be attributed to the specific dietary requirements of citrin patients, including a high-fat diet, or to the metabolic consequences of citrin dysfunction and other factors, such as liver failure, or both. For example, it has been reported that the peroxisome proliferator-activated receptor α, the main regulator of fatty acid oxidation, is downregulated in patients [46]. It is possible that in patients not receiving a high-fat/protein and low-carbohydrate diet, citrin deficiency can have a negative impact on *de novo* lipogenesis required for fat storage. Lipid and carbohydrate metabolism are interlinked because both pathways generate acetyl-CoA, which is converted to citrate. Citrate can be exported from the mitochondrion by the mitochondrial citrate carrier, where it serves as a source of carbon and reducing equivalents for *de novo* lipogenesis (Figure 1). Thus, we hypothesize that impaired sugar metabolism in citrin-deficiency patients could impact negatively on *de novo* lipogenesis in adipose tissues, thus creating the potential for energy crises because of the absence of fat reserves. *de novo* lipogenesis is also important in the third trimester of fetal growth, when myelination of the developing central nervous system and deposition of body fat is required, and its impairment could be a cause of the low birth weight and length. Patients often take pyruvate [47], bypassing glycolysis, and/or medium-chain triglycerides [48] that are degraded by β-oxidation in mitochondria, as sources of metabolic energy (Figure 1).

Another important pathway affected, which is expected to be deregulated in citrin deficiency, is gluconeogenesis [41,49], which primarily occurs in the liver and is one of several mechanisms used by humans to maintain blood glucose levels to avoid hypoglycemia. Gluconeogenesis results in the generation of glucose from non-carbohydrate substrates (Figure 1) such as pyruvate (aerobic conditions) and lactate (anaerobic conditions), glucogenic amino acids, glycerol, and odd-chain fatty acids from the breakdown of lipids. This process is particularly important during overnight fasting because 90% of glucose is then produced by the liver. In support, NICCD and the adaptation stage are indeed characterized by hypoglycemia in many patients. Most glucogenic amino acids were significantly decreased in affected children [50]. In conclusion, patients might not be able to generate enough glucose during periods of fasting and exercise.

Citrin deficiency is classified as a urea cycle disorder [51], and CTLN2 patients typically present with citrullinemia and hyperammonemia [3]. First, a key function of citrin is the export of mitochondrial aspartate that is required for the urea cycle (Figure 1). Second, CTLN2 is also characterized by an unexplained liver-specific decrease in argininosuccinate synthetase, a key enzyme in the urea cycle, which leads to reduced activity [52]. The purpose of the urea cycle is the removal of ammonia produced by deamination reactions in the mitochondrial matrix, and to convert it to urea, which is excreted to achieve detoxification [53]. Ammonia is very toxic to tissues, particularly to neuronal tissues such as the brain, and its accumulation can lead to brain edema and neuropsychiatric symptoms, as sometimes observed in patients. The importance of citrin in the development of citrullinemia and hyperammonemia has been highlighted in a citrin and mitochondrial glycerol-3-phosphate dehydrogenase (mGPD) double-knockout mouse [49]. In this mouse model, application of ornithine in combination with aspartate or alanine effectively suppressed hyperammonemia, lowering blood ammonia levels, by overcoming the defect in aspartate export caused by the citrin knockout [54].

In NICCD and CTLN2, characteristic alterations in blood amino acid levels are also often observed, including increases in citrulline, arginine, methionine, phenylalanine, tyrosine, and threonine levels, as well as the threonine:serine ratio [3]. Citrin functions in the malate-aspartate shuttle that is linked to the tricarboxylic acid (TCA) cycle, a key pathway in amino acid metabolism (Figure 1). In catabolism, isoleucine, methionine, threonine, and valine are converted to succinyl-CoA, whereas phenylalanine and tyrosine are converted to fumarate, both of which are TCA cycle intermediates. The ketogenic amino acids leucine, lysine, phenylalanine, tryptophan, and isoleucine are converted to acetyl-CoA, entering the TCA cycle. Citrin is also crucial for gluconeogenesis, required for the degradation of the glucogenic amino acids alanine, glycine, cysteine, serine, aspartate, and asparagine. Furthermore, the degradation of proline, histidine, arginine, and glutamine leads to glutamate, which is a substrate of citrin. Thus, in citrin deficiency, some deregulation of amino acid metabolism might occur in all stages.

## Sequence, structure, and mechanism of citrin

**Figure 2:**
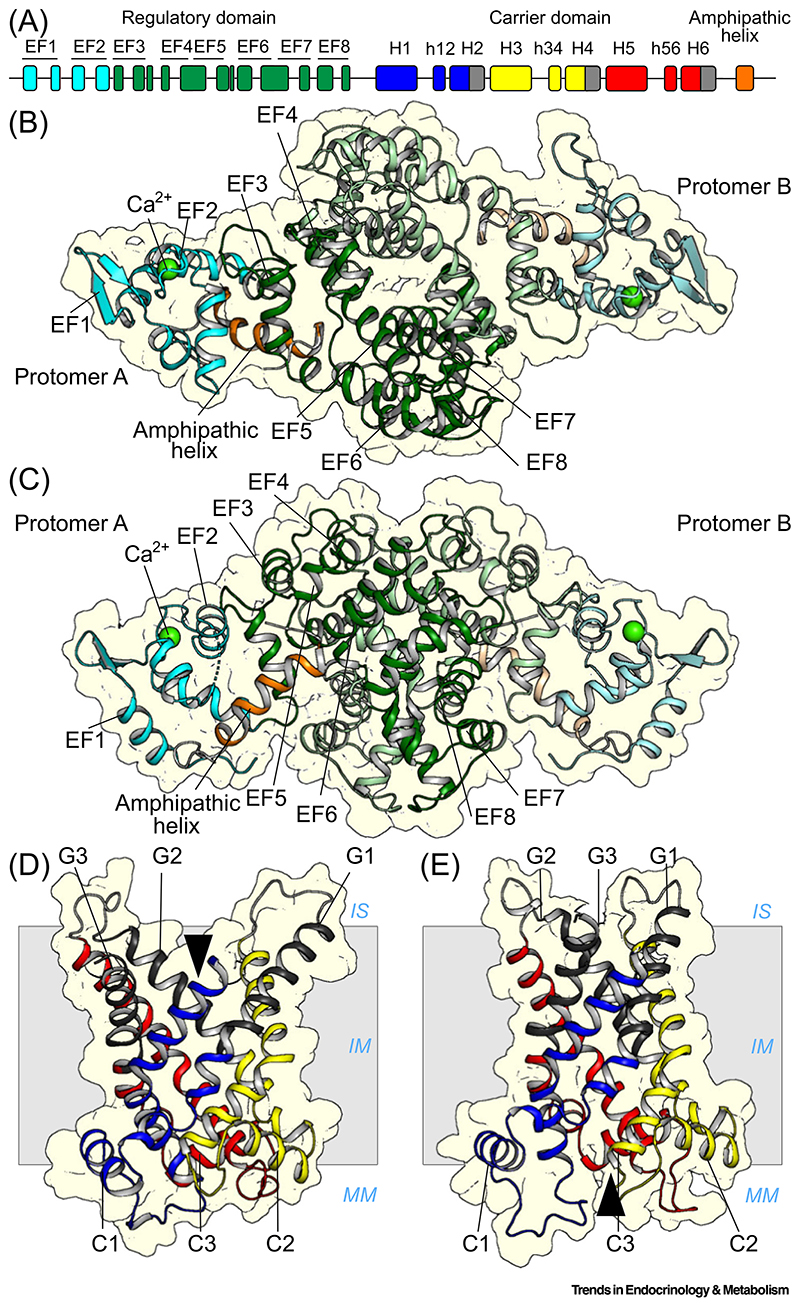
Structural domains of citrin and their functional elements. (A) Structural elements identified in the amino acid sequence of citrin. The N-terminal calcium-regulatory domain has eight EF-hands with the calcium-responsive domain colored in cyan and the static domain, which is involved in dimerization, in green. The carrier domain consists of six transmembrane helices (H1–H6) and three matrix helices (h12, h34, and h56) and is colored by the six functional elements. The three core elements are in primary colors, C1, blue, C2, yellow, and C3, red, whereas the three gate elements G1, G2, and G3 are in gray. The C-terminal domain with the amphipathic helix is colored orange. (B) Cytoplasmic view and (C) lateral view of the N-terminal calcium-regulatory domain with the positions of the eight EF-hands (EF1–8) indicated and the C-terminal amphipathic helix (PDB entry 4P5W), which are colored as in (A) for protomer A and in a lighter shade for protomer B. The domain dimerizes via EF4–8, which together form the static domain (green), whereas EF1–2 form the calcium-responsive domain (cyan), showing a bound calcium ion (green sphere). EF3 is the pivot point of the movements. (D,E) Comparative models of the carrier domain in the cytoplasmic and matrix state, respectively, based on the related structures of the mitochondrial ADP/ATP carrier (PDB entries 4C9Q and 6GCI, respectively), colored by the six functional elements as in (A). The arrowheads indicate the open side of the carrier domain. Abbreviations: IM inner membrane; IS, intermembrane space; MM, mitochondrial matrix; PDB, Protein Data Bank.

**Figure 3:**
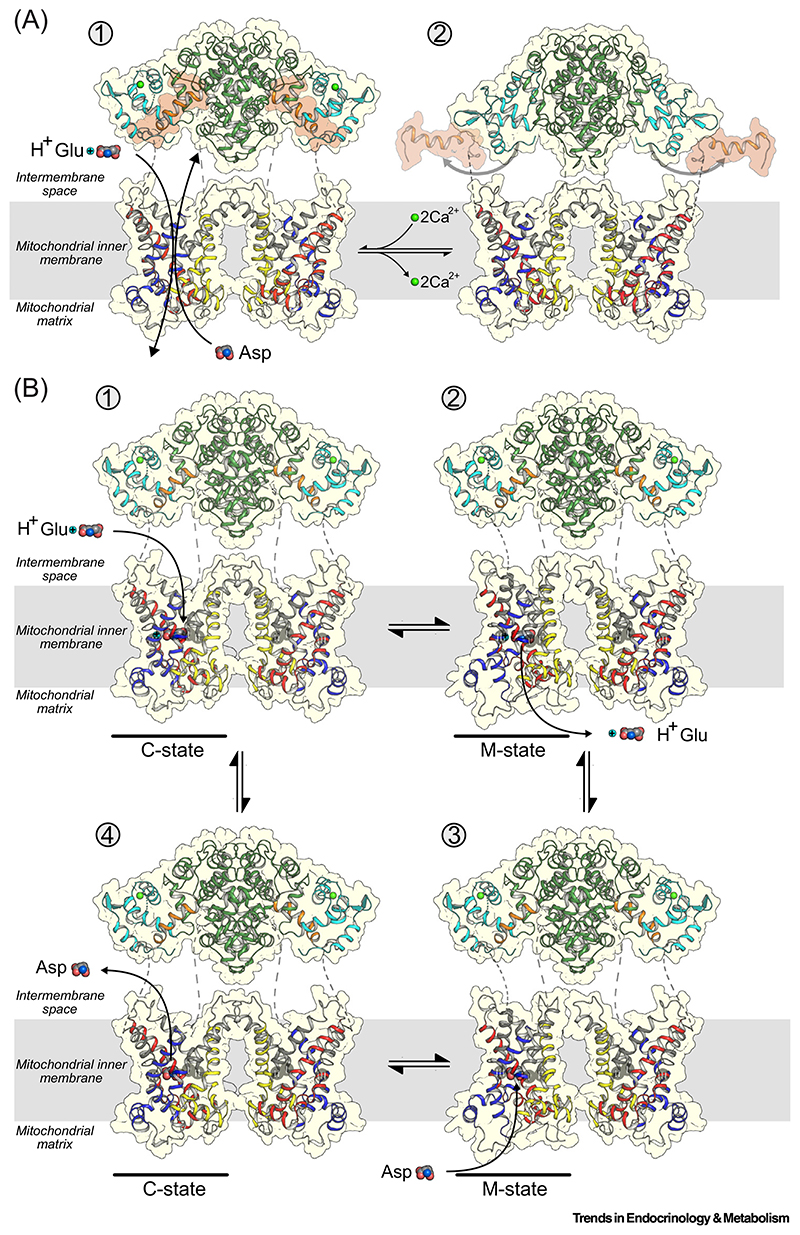
Mechanism of calcium regulation and transport. (A) Stage 1, the calcium-bound state (PDB entry 4P5W), and stage 2, the calcium-free state, based on aralar (PDB entry 4P6O). Upon calcium binding, the amphipathic helix of the C-terminal domain binds to the N-terminal domain, and the carrier domains are able to transport substrates. In the absence of calcium, the mobile unit closes, leading to release of the amphipathic helix, but it is unclear how it leads to inhibition of transport. (B) Representation of four stages of the transport mechanism. Stage 1, glutamate together with a proton from the intermembrane space binds to the binding site of the carrier domain in the cytoplasmic state. Stage 2, binding triggers a conformation change from the cytoplasmic state to the matrix state via an occluded state, after which glutamate and proton leave the binding site and diffuse into the mitochondrial matrix. Stage 3, aspartate binds to the binding site of the carrier domain in the matrix state. Stage 4, binding triggers a conformation change from the matrix to the cytoplasmic state via an occluded state, after which aspartate leaves the binding site and diffuses into the intermembrane space. The structural models are based on the structures of the mitochondrial ADP/ATP carrier in two states (PDB entries 4C9G and 6GCI). The color coding of the domains is the same as in Figure 2. Abbreviations: C-state, cytoplasmic-open conformational state; M-state, matrix-open conformational state; PDB, Protein Data Bank.

Although the genes had been described previously [20,31,55], the molecular identities of the mitochondrial aspartate/glutamate carriers were only confirmed in 2001 [26]. The two human isoforms, citrin and aralar (AGC2 and AGC1, respectively), are the largest members of the SLC25 mitochondrial carrier family [26] and have an unusual three-domain architecture [35]. They have an N-terminal calcium-regulatory domain, which has eight **EF-hand** motifs [38] –helix-loop-helix structural motifs that operate in pairs and change conformation upon binding of calcium ions [38,56]. This is followed by a carrier domain with a structure typical of members of the SLC25 mitochondrial carrier family [26,36,37], which carries out the transport steps. Finally, they have a C-terminal domain comprising an amphipathic α-helix which is a key component of the regulatory mechanism (Figure 2A) [35]. When citrin was purified, it was found to have a protein mass of 148 kDa, twice the molecular mass of 74 kDa encoded by the gene, demonstrating that it is a structural homodimer [35]. The dimerization is mediated by repurposed EF-hands 4–8 of the N-terminal domain, forming a static domain (Figure 2B,C), whereas the carrier domains do not associate [35]. By contrast, EF-hands 1–3 form a calcium-responsive mobile unit (Figure 2B,C). Only EF-hand 2 binds calcium in a preformed binding site, and EF-hands 1 and 2 together are involved in large conformational changes between the calcium-bound and free states (Figure 3A), whereas EF-hand 3 acts as a pivot point [35]. Another remarkable feature is that the C-terminal amphipathic helix is bound to the N-terminal domain in a hydrophobic cleft when the regulatory domain is in a calcium-bound state (Figure 2B,C). In the calcium-free state, conformational changes of the mobile domain close the cleft and release the amphipathic helix, but it is unknown how this leads to inhibition of transport (Figures 2B,C and 3A).

The carrier domains have a similar structure to the mitochondrial ADP/ATP carrier which has been characterized in great detail [36,37,57–61]. They have a threefold pseudo-symmetrical structure with three domains of similar structure [58], reflecting the three homologous sequence repeats [62] (Figure 2D,E). Each domain consists of two transmembrane helices linked by a loop containing a matrix helix, which fold up into a six-helical bundle [57] (Figure 2D,E). The carrier domains cycle between two conformational states in an **alternating access** mechanism: a **matrix state**, where the substrate binding site is open to the mitochondrial matrix, and a **cytoplasmic state**, where it is open to the intermembrane space which is confluent with the cytoplasm (Figure 3B) [36,37,57–61]. When calcium is bound to the regulatory domain, glutamate and a proton bind to their binding sites in the cytoplasmic state of the carrier domain [63–65], after which it converts to the matrix state (Figure 3B) via an **occluded state** that is closed to both sides. The substrates then diffuse into the mitochondrial matrix. Subsequently, mitochondrial aspartate binds and triggers conformational changes, leading to conversion from the matrix state to the cytoplasmic state, again via an occluded state. Aspartate then diffuses into the intermembrane space, completing the **exchange** of substrates across the mitochondrial inner membrane (Figure 3B).

## The wide range of pathogenic variants of citrin

To date, more than 100 different citrin pathogenic variants have been reported, including 18 splicing site mutations, 25 deletion or insertion mutations, 21 nonsense mutations, and 44 missense mutations (Tables S1–S4 in the supplemental information online). We have compiled data on the prevalence of citrin pathogenic alleles in East Asia, the best-studied cases. Recently, in the largest published cohort of 29 364 newborns, 28 *SLC25A13* pathogenic variants were screened and 658 individuals were identified as carriers of mutated alleles [12]. From this study, the carrier rate in China was estimated to be 1:45 [12], which is quite similar to 1:42 in Northern Japan and 1:65 in Southern Japan [10,11,66], and higher than in Thai and Korean populations, 1:90 and 1:110, respectively [12,67]. The pan-ethnicity is explained by migrations and population increases, spreading citrin deficiency not only to different regions in East Asia (Box 1) but also worldwide. In the following, we separate the variants into different categories based on the major genetic changes.

**Figure 4:**
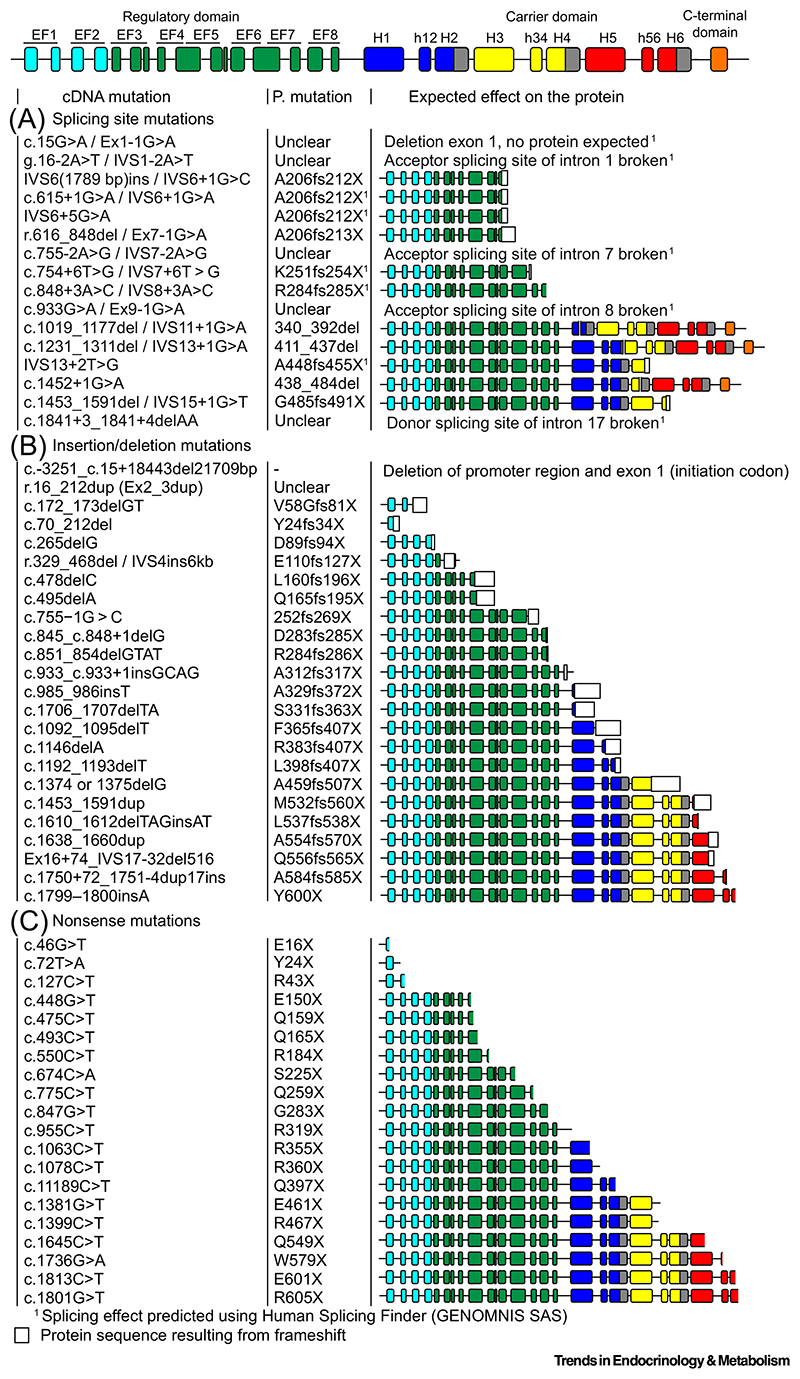
Potential effects of splicing, deletion, insertion, and nonsense mutations on the citrin protein sequence. (A) Splicing site, (B) insertion/deletion, and (C) nonsense variants. The color coding is the same as in Figure 2. The effects of the splicing site mutations were predicted using Human Splicing Finder [76]. It cannot be excluded that the produced mRNA is subject to nonsense-mediated mRNA decay mechanisms, leading to deficient expression [69,70]. The column title ‘P. mutation’ refers to the consequences on the protein level.

Alleles containing splicing site pathogenic mutations are the first (48% in Japan and 33%in Korea) and second (24% in China) most prevalent mutant alleles found in East Asia (Table S1 and Figure 4A) [10,12,68]. The list of 18 reported splicing site variants highlights that 50% of them occur in the intervening sequence 6 (1.8 kb) and 7 (1.7 kb) and most create a premature stop codon. Exon skipping in variants IVS11+1G>A, IVS13+1G>A, and c.1452+1G>A does not create a premature stop codon, but could generate a frame-shifted protein (Figure 4A).

Insertion or deletion mutations have been found in all parts of the citrin gene (Table S2 and Figure 4B). However, three mutations can be distinguished by their high prevalence: c.851_854delGTAT, c.1638_1660dup, and IVS16ins3kb (c.1750+72_1751-4dup17ins). The c.851_854delGTAT mutation is found in >50% of patients in China and represents 60% in terms of allele frequency. It is also found in ~30% of patients in Japan and Korea [10,68].

Genes with nonsense mutations are less prevalent than others (Table S3 and Figure 4C), representing ~4% in China. The most common nonsense mutations in that country are R467X (~1.5%), Q259X (~1%), R319X (~1%), and R184X (~0.5%) (Table S3). However, in Japan and Korea, the prevalence of S225X is much higher, up to 6% and 9% of all patients, respectively. In the case of all splice site, deletion, insertion, and nonsense mutations, the mRNA produced could be subject to nonsense-mediated mRNA decay mechanisms, abolishing expression altogether [69].

**Figure 5:**
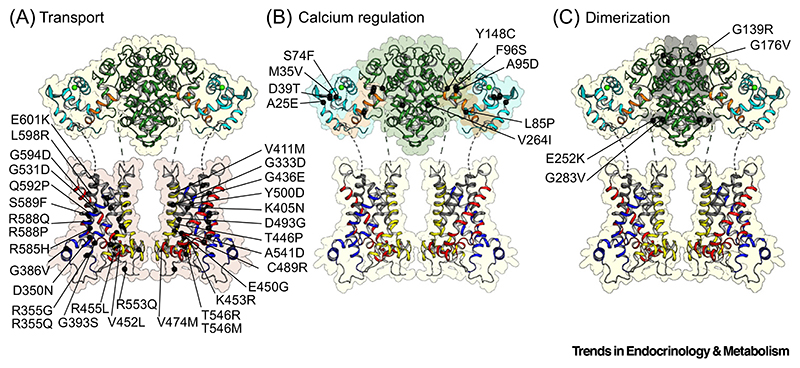
Locations of the different missense mutations in citrin. The positions of all mutations are indicated by black circles. (A) Mutations in the carrier domain of citrin (light pink). (B) Mutations related to the calcium-regulation domain. The flexible parts (cyan), the static parts (forest green), and the C-terminal amphipathic helix (orange) of the regulatory domain are highlighted. (C) Mutations located in the dimerization interface (gray). The color coding of the domains is the same as in Figure 2.

The last category, the missense mutations, represent 20% of all patients found in the Chinese population (Table S4). E252K, G393S, V411M, and A541D are the most common mutations. To date, 44 missense mutations have been identified, of which 70% (30 total) are located in the carrier domains (Figure 5A), whereas 30% (14 of total) are located in the regulatory domain, possibly affecting transport or calcium regulation, respectively (Figure 5B,C).

Pathogenic alleles have been found in several combinations in heterozygous patients, which comprise 53%, whereas homozygotes represent 47%, but 42% of all homozygotes carry the mutation c.851_854delGTAT. The combination of different alleles in compound heterozygotes might have important consequences for the function of citrin, given the fact that it forms dimers [35].

## Predicted effects of mutations on the structure and function of citrin

Before predicting the effect of the various pathogenic mutations, the first consideration is their biogenesis – which involves all steps required for maturation, such as expression, targeting, insertion, folding, and dimerization. The fate of most disease variants in biogenesis is currently unknown, but could affect their expression levels and subcellular localization. For example, the expression of splice site mutations, insertions, deletions, and nonsense mutations might be restricted by quality-control mechanisms controlling the integrity of the transcriptome, such as nonsense-mediated mRNA decay [69,70]. The second consideration is the combination of the two alleles found in each patient. In homozygotes, the presence of only one allele type should result in homodimers, provided that the mutations do not disrupt the dimer interface and that the variant is targeted to mitochondria. In compound heterozygotes, the situation is more complex because two variants could be expressed in some cases, potentially giving rise to mixed populations of affected homo- and heterodimers. We attempt later to predict the consequences of different pathogenic mutations in a homozygosity context, focusing on the different functional domains.

The vast majority of the splicing site mutations, insertions, deletions, and nonsense mutations result in complete or partial deletion of the carrier domains (Figure 4) if expressed. However, as mentioned earlier, most of the proteins might not be produced. Even if their biogenesis was normal, these variants would not be able to transport substrates because that function requires the structural integrity of the carrier domains (Figure 2D,E). Even mutations resulting in relatively small C-terminal truncations, such as c.1813C>T, c.1801G>T, c.1799-1800insA, and c.1750+72_1751-4dup17ins, could disrupt transmembrane helix 6, leading to loss of transport function (Figure 4). In addition, these variants would also lack the C-terminal domain with the amphipathic helix, a key part of the calcium-regulatory mechanism. It is unknown, however, whether some of these alleles can be expressed as truncated proteins and whether their presence would interfere with the function of a second, active or partially active protomer within dimers in the case of compound heterozygotes or whether they would interfere with other cellular processes, such as mitochondrial protein import.

Other mutations affecting the carrier domains are missense mutations (Figure 5A), which are not expected to be subject to mRNA control mechanisms. Interestingly, only four missense mutations have been associated with adult-onset CTNL2, the severe form of the disease. It is most likely that most of these variants are expressed and targeted to the mitochondrial inner membrane, with the possible exception of those that are critical for the structural integrity of citrin. Normal expression levels have been reported for mutant G437E in fibroblasts of a heterozygous patient [13] and for A25E in a homozygous patient [18], but most studies do not address the expression of missense variants in patients or in mammalian cell models. For the missense variants, which are expressed and localized properly, the transport and regulatory activities need to be experimentally determined, but this has not been done for the majority of disease variants. Several missense mutations in the carrier domains are found in positions critical for their structure and function [37,61,71]. Even though the substrate binding site is unique, many other features are universally conserved among SLC25 mitochondrial carriers, such as a **matrix salt-bridge network** [36,57,72], **glutamine braces** [36], the **Pro/Ser-kink** [36,57], **cytoplasmic salt-bridge network** [36,37,73,74], **tyrosine braces** [37], as well as **gate** and small residues that are required for conformational state interconversions [37,61,71]. A previous review looked into the pathogenic mutations in citrin in the context of these key features [71]. The effect of substitutions could be dependent on the type of replacement, raising the possibility of different functional and phenotypic consequences. Overall, missense variants are less likely to show impaired biogenesis and are more likely to be active to some extent.

Some splice site mutations, insertions, deletions, and nonsense mutations would also impact on the function of the regulatory domain, if expressed (Figure 4), but it is likely that biogenesis is compromised, potentially leading to issues with other cellular processes. However, 13 missense mutations have been identified in the regulatory domain. Nine are found either in the mobile or static unit (Figure 5B), potentially affecting calcium regulation, whereas another four are located in the dimerization interface, potentially affecting dimer formation (Figure 5C) [35].

Interestingly, no missense variants have been found in the C-terminal amphipathic helix, even though it is expected to be critical for regulation.

## Concluding remarks

Although citrin deficiency is now recognized as a pan-ethnic disease, affecting hundreds of thousands of patients to variable degrees, disease awareness remains very limited. Moreover, the molecular and cellular mechanisms underlying the disease are poorly understood and its metabolic consequences have not been fully elucidated (reviewed in [3,8,48,75]). In the past few years, the Citrin Foundation (https://citrinfoundation.org) has initiated a global effort to increase awareness and support interdisciplinary research to elucidate the basic molecular and cellular mechanisms underlying citrin deficiency and to develop new diagnostic and therapeutic strategies.

The current understanding of the disease pathophysiology is largely based on the work of Saheki and colleagues, who first identified the causative effect of the AGC2 mutations and the involvement of ASS1 enzyme levels in late onset of the disease (CTLN2) [1,2,8]. However, many aspects of this disease remain poorly understood (see Outstanding questions).

One important question to answer is whether there is a relationship between citrin mutations and the diverse phenotypes. Different pathogenic variants might have different effects on mitochondrial physiology and consequently on disease progression and severity. The first obstacle is the lack of complete, accurate, and reliable documentation of the reported citrin mutations and associated disease phenotypes as groups use different protocols for their analysis. For example, genotypic analysis varies from full exome sequencing to only the detection of specific high-frequency mutations, opening the possibility that additional, unidentified mutations might have been missed in the population or that there are more biallelic mutations than currently reported. Second, expression levels of the disease variants have only been assessed and reported anecdotally [13,18], and without mitochondrial localization data. Third, for the vast majority of the reported mutations, no functional analysis has been performed. What makes the situation even more complex is the fact that the majority of patients are compound heterozygotes whereas citrin is dimeric, making assessment at the molecular and cellular levels even more challenging.

These challenges need to be overcome by establishing robust and consistent clinical protocols as well as appropriate model systems. Assessment of citrin levels in patients could be key to any subsequent analysis, although this is usually hampered by the lack of biopsy samples. Appropriate cellular or animal model systems to study the expression, localization, and effects of different pathogenic variants on mitochondrial physiology are currently missing. To that end, cellular models as well as new mouse models that better represent the genotypic and phenotypic characteristics need to be developed, including conditional knockout and knock-in mutants. Robust metabolic and bioenergetic profiling will be key to understanding several features of the disease and to plan therapeutic strategies. Development of better imaging techniques, including super-resolution microscopy and electron tomography, will be key to addressing the effects of pathogenic variants on mitochondrial ultrastructure and dynamics.

A factor of major importance is to assess the function of the different citrin pathogenic variants, when expressed. Thus far, the transport activity of the aspartate/glutamate carrier has been estimated either indirectly by measuring the activity of the malate-aspartate shuttle [40] or by transport assays using refolded protein in liposomes [26]. However, protein refolding is inefficient, requiring the correct folding of three domains and dimerization, and thus, the calcium-regulatory function was not assessed [26]. Thus, different methods need to be established to obtain purified wild-type and mutant proteins in their folded state and to establish quality-control assessments for protein integrity before comparisons of transport and regulatory activities of variants are attempted.

Structural analysis of citrin can also provide a wealth of information on its function and even allow predictions of the functional effects of pathogenic mutations. We have listed here all the reported citrin pathogenic variants and used structural models of full-length citrin to locate each mutation. Clearly, these predictions require rigorous experimental validation, but they can serve as a guideline for future studies.

Citrin deficiency is an understudied, underestimated, and complex disease, causing multiple effects on physiology. Combined interdisciplinary efforts and increased awareness within the scientific and medical communities are necessary to solve the outstanding questions and to provide reliable therapeutic strategies and interventions.

## Supplementary Material

Supplementary Material

## Highlights

Citrin deficiency is caused by the absence or dysfunction of the mitochondrial aspartate/glutamate carrier 2 (AGC2), also called citrin, which imports glutamate together with a proton into mitochondria and exports aspartate.

The disease is highly prevalent and panethnic with highest occurrence in East Asia and affects the function of the malate-aspartate shuttle, energy metabolism, and the urea cycle, causing a range of highly variable disease symptoms.

Recent structural work has shown that citrin is a structural dimer and has highlighted the different functional elements that are important for its calcium-regulatory and transport mechanism.

The large number of splicing, insertion, deletion, nonsense, and missense mutations can now be put in a structural context, which facilitates the evaluation of their impact on the mechanism and function of citrin in the different disease phenotypes.

## Box 1 Historical and geographical distribution of citrin-deficiency disease variants

The geographical distributions of the pathogenic mutations tell the history of the East Asian populations from the Neolithic period to the modern age [66,77,78]. Indeed, the modern Chinese population comes from Northern and Southern populations [79–81], identified during the Early Neolithic times (~8400 years ago), which were separated by the Yangtze River (Figure IA). In China, there are four main citrin-deficiency mutations: c.851-854delGTAT (I), IVS16ins3kb (XIX), c.1638_1660dup (III), and IVS6+5G>A (X) (Figure IB – China). The c.1638_1660dup and IVS6+5G>A mutations are uniformly distributed in various regions of China, in contrast to the c.851-854delGTAT and IVS16ins3kb mutations which vary with location. The c.851-854delGTAT mutation is found in >50% of the citrin-deficiency carriers in South China [82], but the prevalence decreases gradually moving to the North, whereas the IVS16ins3kb mutation has the opposite tendency [12,66,78,82] (Figure IB: Northern, Border, and Southern regions). Outside China, the frequency of c.851-854delGTAT is the highest in the Southern regions of Asia, such as Taiwan [83], Thailand [67], Singapore [84], and Vietnam [85], and is high in South Korea [10,66,68] and Japan [10,66,86,87] (Figure IB). Interestingly, the c.851-854delGTAT mutation follows exactly the different expansion routes of the Southeastern populations of China (Figure IA). Indeed, during the Neolithic period and Bronze Age, the Southeast population started multiple migration waves to South Asia [88,89] and Japan [89,90], but to a lesser extent to Korea [90]. The current proportions of the Korean population show that it mixed mainly with populations from Northeast Asia [81], as is evident from the IVS16ins3kb mutation. In contrast to South Asia, the IVS16ins3kb mutation is as common as the c.851-854delGTAT mutation in the Northern regions of China [12,66,78,82] and South Korea [10,66,68], but it is almost absent from Japan [10,66,86,87]. Indeed, ancestors from Northeast Asia played a more important role in the migration to Southeast Asia than ancestors from Southeast Asia to the North [81]. Thus, greater North–South migration explains why the c.851-854delGTAT mutation is much less prevalent in the Northeast than in the Southeast. The IVS11+1G>A (II) and S225X (IV) mutations are almost exclusively found in Japan [10,66,86,87] and South Korea [10,66,68]. The mutation IVS11+1G>A is largely absent in Korea, indicating that it occurred before the split of Korea and Japan [66] about 1200–3600 years ago [91].

## Outstanding questions

How can the diverse phenotypes of citrin deficiency be explained?

Why does CTLN2, the late-onset, severe form of the disease, develop in only a subset of patients?

Do different mutations correlate with disease phenotypes and CTLN2 onset?

What is the impact of the different mutations on mitochondrial and cellular physiology?

Are the various pathogenic variants of citrin expressed in patients and targeted to mitochondria?

Could they sustain some level of transport or regulatory activity?

What is the metabolic fingerprint of patients with different mutations?

Do citrin-deficiency patients have different levels of energy deficits?

How does expression or function of other proteins, such as aralar and ASS1, interfere with citrin-deficiency phenotypes?

Which mechanisms underlie the development of fatty liver disease, non-alcoholic steatohepatitis, and liver cancer in citrin-deficiency patients?

## glossary

Alternating access: a transport mechanism used by most secondary active transporters in which the substratebinding site is exposed to one or other side of the membrane in an alternating fashion.
Amphipathic helix: an α-helix that has a hydrophobic side and a hydrophilic side.
Cytoplasmic salt-bridge network: three ionic interactions between the negatively and positively charged residues of the conserved [YF][DE]xx[KR] motifs, linking the even-numbered α-helices of SLC25 proteins in the matrix state.
Cytoplasmic state: a conformational state of an SLC25 carrier in which its central substrate-binding site is accessible to the mitochondrial intermembrane space, which is confluent with the cytosol.
EF-hand: a helix-loop-helix structural motif found in proteins that can bind calcium ions, leading to a conformational change.
Exchange: secondary active transport process that exchanges two molecules across a biological membrane.
Gate: a set of amino acid residues of a transport protein, which in one conformational state block access of substrate from one side of the membrane to its binding site and opens in the other conformation state.
Glutamine brace: amino acid residues lying on the matrix side of the matrix saltbridge networks, frequently glutamines, which can form additional hydrogen bonds that help to stabilize the matrix salt-bridge network.
Matrix salt-bridge network: three ionic interactions between the negatively and positively charged residues of the conserved Px[DE]xx[KR] signature motifs, frequently charged, linking the odd-numbered α-helices of SLC25 proteins in the cytoplasmic state.
Matrix state: a conformational state of an SLC25 carrier in which its central substrate-binding site is accessible to the mitochondrial matrix.
Occluded state: an intermediate state in the transport cycle in which both sides of the carrier are closed, preventing uncontrolled movement of molecules and ions across the membrane.
Pro/Ser-kink: structural kink in the odd-numbered α-helices of SLC25 proteins caused by disruption of the hydrogen bonding of the α-helix by proline or serine residues.
Tyrosine brace: tyrosine residues on the matrix side of the cytoplasmic saltbridge networks, which can form hydrogen bonds with negatively charged residues of the neighboring domain via their OH groups, helping to stabilize the network.

## References

[1] Saheki T (2002). Pathogenesis and pathophysiology of citrin (a mitochondrial aspartate glutamate carrier) deficiency. Metab Brain Dis.

[2] Saheki T, Kobayashi K (2002). Mitochondrial aspartate glutamate carrier (citrin) deficiency as the cause of adult-onset type II citrullinemia (CTLN2) and idiopathic neonatal hepatitis (NICCD). J Hum Genet.

[3] Okano Y (2019). Current treatment for citrin deficiency during NICCD and adaptation/compensation stages: strategy to prevent CTLN2. Mol Genet Metab.

[4] Song YZ (2009). Failure to thrive and dyslipidemia caused by citrin deficiency: a novel clinical phenotype. Zhongguo Dang Dai Er Ke Za Zhi.

[5] Saheki T (2010). Citrin deficiency and current treatment concepts. Mol Genet Metab.

[6] Saheki T, Song YZ, Adam MP (2005). GeneReviews.

[7] Hayasaka K, Numakura C (2018). Adult-onset type II citrullinemia: current insights and therapy. Appl Clin Genet.

[8] Saheki T (2020). AGC2 (Citrin) deficiency – from recognition of the disease till construction of therapeutic procedures. Biomolecules.

[9] Morrow G, Barness LA (1968). Citrullinemia. Pediatrics.

[10] Tabata A (2008). Identification of 13 novel mutations including a retrotransposal insertion in SLC25A13 gene and frequency of 30 mutations found in patients with citrin deficiency. J Hum Genet.

[11] Kikuchi A (2012). Simple and rapid genetic testing for citrin deficiency by screening 11 prevalent mutations in SLC25A13. Mol Genet Metab.

[12] Lin Y (2020). Combining newborn metabolic and genetic screening for neonatal intrahepatic cholestasis caused by citrin deficiency. J Inherit Metab Dis.

[13] Fiermonte G (2011). A new Caucasian case of neonatal intrahe-patic cholestasis caused by citrin deficiency (NICCD): a clinical, molecular, and functional study. Mol Genet Metab.

[14] Hutchin T (2009). Neonatal intrahepatic cholestasis caused by citrin deficiency (NICCD) as a cause of liver disease in infants in the UK. J Inherit Metab Dis.

[15] Ben-Shalom E (2002). Infantile citrullinemia caused by citrin deficiency with increased dibasic amino acids. Mol Genet Metab.

[16] Vitoria I (2013). Citrin deficiency in a Romanian child living in Spain highlights the worldwide distribution of this defect and illustrates the value of nutritional therapy. Mol Genet Metab.

[17] Avdjieva-Tzavella DM (2014). First Bulgarian case of citrin deficiency caused by one novel and one recurrent mutation in the SLC25A13 gene. Genet Couns.

[18] Dimmock D (2009). Citrin deficiency, a perplexing global disorder. Mol Genet Metab.

[19] Pinto A (2020). Dietary management, clinical status and outcome of patients with citrin deficiency in the UK. Nutrients.

[20] Kobayashi K (1999). The gene mutated in adult-onset type II citrullinaemia encodes a putative mitochondrial carrier protein. Nat Genet.

[21] Azzi A (1967). Penetration of the mitochondrial membrane by glutamate and aspartate. Biochem Biophys Res Commun.

[22] LaNoue KF (1974). Evidence for electrogenic aspartate transport in rat liver mitochondria. Arch Biochem Biophys.

[23] LaNoue KF (1974). Energy-driven aspartate efflux from heart and liver mitochondria. J Biol Chem.

[24] Dierks T (1988). Reaction mechanism of the reconstituted aspartate/glutamate carrier from bovine heart mitochondria. Biochim Biophys Acta.

[25] Dierks T, Kramer R (1988). Asymmetric orientation of the reconstituted aspartate/glutamate carrier from mitochondria. Biochim Biophys Acta.

[26] Palmieri L (2001). Citrin and aralar1 are Ca^2+^-stimulated as-partate/glutamate transporters in mitochondria. EMBO J.

[27] LaNoue KF, Schoolwerth AC (1979). Metabolite transport in mitochondria. Annu Rev Biochem.

[28] Meijer AJ (1978). Interrelationships between gluconeogenesis and ureogenesis in isolated hepatocytes. J Biol Chem.

[29] Chappell JB (1968). Systems used for the transport of substrates into mitochondria. Br Med Bull.

[30] Borst P (2020). The malate-aspartate shuttle (Borst cycle): how it started and developed into a major metabolic pathway. IUBMB Life.

[31] del Arco A, Satrustegui J (1998). Molecular cloning of Aralar, a new member of the mitochondrial carrier superfamily that binds calcium and is present in human muscle and brain. J Biol Chem.

[32] Wang D (2019). A deep proteome and transcriptome abundance atlas of 29 healthy human tissues. Mol Syst Biol.

[33] Wibom R (2009). AGC1 deficiency associated with global cerebral hypomyelination. N Engl J Med.

[34] Falk MJ (2014). AGC1 deficiency causes infantile epilepsy, abnormal myelination, and reduced N-acetylaspartate. JIMD Rep.

[35] Thangaratnarajah C (2014). Calcium-induced conformational changes of the regulatory domain of human mitochondrial aspartate/glutamate carriers. Nat Commun.

[36] Ruprecht JJ (2014). Structures of yeast mitochondrial ADP/ATP carriers support a domain-based alternating-access transport mechanism. ProcNatlAcadSciUSA.

[37] Ruprecht JJ (2019). The molecular mechanism of transport by the mitochondrial ADP/ATP carrier. Cell.

[38] Contreras L (2007). Ca^2+^activation kinetics of the two aspartate-glutamate mitochondrial carriers, aralar and citrin: role in the heart malate-aspartate NADH shuttle. J Biol Chem.

[39] Iacobazzi V (1992). Sequences of the human and bovine genes for the mitochondrial 2-oxoglutarate carrier. DNA Seq.

[40] Jalil MA (2005). Reduced N-acetylaspartate levels in mice lacking aralar, a brain- and muscle-type mitochondrial aspartateglutamate carrier. J Biol Chem.

[41] Sinasac DS (2004). Slc25a13-knockout mice harbor metabolic deficits but fail to display hallmarks of adult-onset type II citrullinemia. Mol Cell Biol.

[42] Saheki T (2017). Oral aversion to dietary sugar, ethanol and glycerol correlates with alterations in specific hepatic metabolites in a mouse model of human citrin deficiency. Mol Genet Metab.

[43] Mracek T (2013). The function and the role of the mitochondrial glycerol-3-phosphate dehydrogenase in mammalian tissues. Biochim Biophys Acta.

[44] Okamoto M (2021). Food preferences of patients with citrin deficiency. Nutrients.

[45] Okano Y (2021). Analysis of daily energy, protein, fat, and carbohydrate intake in citrin-deficient patients: towards prevention of adult-onset type II citrullinemia. Mol Genet Metab.

[46] Komatsu M (2015). Steatogenesis in adult-onset type II citrullinemia is associated with down-regulation of PPARα. Biochim Biophys Acta.

[47] Mutoh K (2008). Treatment of a citrin-deficient patient at the early stage of adult-onset type II citrullinaemia with arginine and sodium pyruvate. J Inherit Metab Dis.

[48] Hayasaka K (2021). Metabolic basis and treatment of citrin deficiency. J Inherit Metab Dis.

[49] Saheki T (2007). Citrin/mitochondrial glycerol-3-phosphate dehydrogenase double knock-out mice recapitulate features of human citrin deficiency. J Biol Chem.

[50] Miyazaki T (2019). Serum amino acid profiling in citrin-deficient children exhibiting normal liver function during the apparently healthy period. JIMD Rep.

[51] Ah Mew N, Adam MP (2003). GeneReviews.

[52] Saheki T (1987). Hereditary disorders of the urea cycle in man: biochemical and molecular approaches. Rev Physiol Biochem Pharmacol.

[53] Krebs HA (1947). Urea synthesis in mammalian liver. Nature.

[54] Saheki T (2019). Pivotal role of inter-organ aspartate metabolism for treatment of mitochondrial aspartate-glutamate carrier 2 (citrin) deficiency, based on the mouse model. Sci Rep.

[55] Del Arco A (2000). Characterization of a second member of the subfamily of calcium-binding mitochondrial carriers expressed in human non-excitable tissues. Biochem J.

[56] Lasorsa FM (2003). Recombinant expression of the Ca^2+^-sensitive aspartate/glutamate carrier increases mitochondrial ATP production in agonist-stimulated Chinese hamster ovary cells. J Biol Chem.

[57] Pebay-Peyroula E (2003). Structure of mitochondrial ADP/ ATP carrier in complex with carboxyatractyloside. Nature.

[58] Kunji ERS, Harding M (2003). Projection structure of the atractyloside-inhibited mitochondrial ADP/ATP carrier of Saccharomyces cerevisiae. J Biol Chem.

[59] Ruprecht JJ, Kunji ERS (2019). Structural changes in the transport cycle of the mitochondrial ADP/ATP carrier. Curr Opin Struct Biol.

[60] Kunji ERS, Ruprecht JJ (2020). The mitochondrial ADP/ ATP carrier exists and functions as a monomer. Biochem Soc Trans.

[61] Ruprecht JJ, Kunji ERS (2020). The SLC25 mitochondrial carrier family: structure and mechanism. Trends Biochem Sci.

[62] Saraste M, Walker JE (1982). Internal sequence repeats and the path of polypeptide in mitochondrial ADP/ATP translocase. FEBS Lett.

[63] Kunji ERS, Robinson AJ (2006). The conserved substrate binding site of mitochondrial carriers. Biochim Biophys Acta.

[64] Robinson AJ, Kunji ERS (2006). Mitochondrial carriers in the cytoplasmic state have a common substrate binding site. Proc Natl Acad Sci U S A.

[65] Kunji ERS, Robinson AJ (2010). Coupling of proton and substrate translocation in the transport cycle of mitochondrial carriers. Curr Opin Struct Biol.

[66] Lu YB (2005). Frequency and distribution in East Asia of 12 mutations identified in the SLC25A13 gene of Japanese patients with citrin deficiency. J Hum Genet.

[67] Wongkittichote P (2013). Screening of SLC25A13 mutation in the Thai population. World J Gastroenterol.

[68] Oh SH (2017). Biochemical and molecular characteristics of citrin deficiency in Korean children. J Hum Genet.

[69] Brogna S, Wen J (2009). Nonsense-mediated mRNA decay (NMD) mechanisms. Nat Struct Mol Biol.

[70] Kurosaki T (2019). Quality and quantity control of gene expression by nonsense-mediated mRNA decay. Nat Rev Mol Cell Biol.

[71] Kunji ERS (2020). The SLC25 carrier family: important transport proteins in mitochondrial physiology and pathology. Physiology.

[72] Nelson DR (1998). Highly conserved charge-pair networks in the mitochondrial carrier family. J Mol Biol.

[73] Robinson AJ (2008). The mechanism of transport by mitochondrial carriers based on analysis of symmetry. Proc Natl Acad Sci U S A.

[74] King MS (2016). Formation of a cytoplasmic salt bridge network in the matrix state is a fundamental step in the transport mechanism of the mitochondrial ADP/ATP carrier. Biochim Biophys Acta.

[75] Saheki T (2005). Metabolic derangements in deficiency of citrin, a liver-type mitochondrial aspartate-glutamate carrier. Hepatol Res.

[76] Desmet FO (2009). Human Splicing Finder: an online bioinformatics tool to predict splicing signals. Nucleic Acids Res.

[77] Song YZ (2011). Genotypic and phenotypic features of citrin deficiency: five-year experience in a Chinese pediatric center. Int J Mol Med.

[78] Chen R (2013). Different regional distribution of SLC25A13 mutations in Chinese patients with neonatal intrahepatic cholestasis. World J Gastroenterol.

[79] Zhao T, Dao Lee T (1989). Gm and Km allotypes in 74 Chinese populations: a hypothesis of the origin of the Chinese nation. Hum Genet.

[80] Du R (1997). Genetic distances between Chinese populations calculated on gene frequencies of 38 loci. Sci China C Life Sci.

[81] Yang MA (2020). Ancient DNA indicates human population shifts and admixture in northern and southern China. Science.

[82] Lin WX (2016). Molecular diagnosis of pediatric patients with citrin deficiency in China: SLC25A13 mutation spectrum and the geographic distribution. Sci Rep.

[83] Lin J-T (2011). High resolution melting analysis for the detection of SLC25A13 gene mutations in Taiwan. Clin Chim Acta.

[84] Bylstra Y (2019). Population genomics in South East Asia captures unexpectedly high carrier frequency for treatable inherited disorders. Genet Med.

[85] Nguyen TMH (2015). Most common SLC25A13 mutation in 695 Vietnamese patients with NICCD. Ann Transl Med.

[86] Kobayashi K (2003). Screening of nine SLC25A13 mutations: their frequency in patients with citrin deficiency and high carrier rates in Asian populations. Mol Genet Metab.

[87] Yamaguchi N (2002). Screening of SLC25A13 mutations in early and late onset patients with citrin deficiency and in the Japanese population: identification of two novel mutations and establishment of multiple DNA diagnosis methods for nine mutations. Hum Mutat.

[88] Lipson M (2018). Ancient genomes document multiple waves of migration in Southeast Asian prehistory. Science.

[89] McColl H (2018). The prehistoric peopling of Southeast Asia. Science.

[90] Kim J (2020). The origin and composition of Korean ethnicity analyzed by ancient and present-day genome sequences. Genome Biol Evol.

[91] Wang Y (2018). Genetic structure, divergence and admixture of Han Chinese, Japanese and Korean populations. Hereditas.

