# Supplementary material for: Pathogenic variants of the mitochondrial aspartate/glutamate carrier causing citrin deficiency
Source: Trends Endocrinol Metab. Author manuscript; Available in PMC 2023 Feb 24. (PMC7614230; doi:10.1016/j.tem.2022.05.002)
Supplement: Supplementary Material [file EMS164660-supplement-Supplementary_Material.docx]

**Supplementary information**

**Title:** Pathogenic variants of the mitochondrial aspartate/glutamate carrier causing citrin deficiency

**Authors:** Sotiria Tavoulari^†^, Denis Lacabanne^†^, Chancievan Thangaratnarajah, and Edmund R.S. Kunji

**Institution:** Medical Research Council Mitochondrial Biology Unit, University of Cambridge, The Keith Peters Building, Cambridge Biomedical Campus, Hills Road, Cambridge, CB2 0XY United Kingdom.

***Table 1.*** *List of known splice site mutations involved in citrin deficiency with their allele frequencies among general populations (column Carriers) and citrin-deficiency patients (column Patients). The frequencies in bold have been estimated based on cohorts from China (C), Japan (J) or Korea (K), reported in the literature. In case of several citrin deficiency patient cohorts, a mean of all the samples was calculated (the cohorts are indicated in references). The frequencies in regular fonts represent an estimation of the carrier prevalence in China based on the carrier (heterozygote) rate of 1/45 from a cohort of 29,364 newborns [12]. The mutations follow the HGVS nomenclature, their other names (most frequently used in literature) are indicated in brackets.*

| **Exon** | **Splicing site mutations** | **Amino acids** | **Frequency** | | **References** |
| --- | --- | --- | --- | --- | --- |
|  |  |  | **Carriers** | **Patients** |  |
| Ex1 | c.15G>A (Ex1-1G>A) | Unclear |  | **~1/200 (J)** | [10,92] |
| IVS1 | c.16-2A>T (IVS1-2A>T) | Unclear |  |  | [93] |
| IVS2 | c.69+5G>A | 6_23del |  |  | [94] |
| IVS6 | c.615+1G>C (IVS6+1G>C) | A206fs212X |  |  | [10] |
| IVS6 | c.615+1G>A (IVS6+1G>A) | A206fs212X1 | ~1/34000 (C) | **~1/750 (C)** | [78,95] |
| IVS6 | c.615+5G>A (IVS6+5G>A) | A206fs212X1 | **~1/320 (C)** | **~1/10 (C)** | [10,12,66,82,93] |
| Ex7 | c.754G>A (r.616_848del) | A206fs213X |  |  | [12,82,93,96] |
| IVS7 | c.755-2A>G (IVS7-2A>G) | Unclear |  |  | [97] |
| IVS7 | c.754+6T>G (IVS7+6T>G) | K251fs254X1 |  |  | [92] |
| IVS8 | c.848+3A>C (IVS8+3A>C) | R284fs285X1 |  |  | [98] |
| Ex9 | c.933G>A (Ex9-1G>A) | Unclear |  |  | [93] |
| IVS11 | c.1019_1177del (IVS11+1G>A) | 340_392del |  | **~1/3 (J)** | [12,78,82,93,99,100] |
| IVS13 | c.1231_1311del (IVS13+1G>A) | 411_437del |  | **~1/10 (J)** | [10,99] |
| IVS13 | c.1311+2T>G (IVS13+2T>G) | A448fs455X1 |  |  | [10] |
| Ex14 | c.1452+1G>A | 438_484del | ~1/70000 (C) | **~1/1550 (C)** | [82] |
| Ex14 | c.1453-1G>A | G485fs507X |  |  | [94] |
| IVS15 | c.1453_1591del (IVS15+1G>T) | G485fs491X |  | **~1/650 (J)** | [10] |
| Ex17 | c.1841+3_1841+4del | Unclear |  |  | [101] |

***Table 2.*** *List of known insertions and deletion mutations involved in citrin deficiency with their frequencies. Markings are as in Table 1.*

| **Exon** | **Insertion/deletion mutations** | **Amino acids** | **Frequency** | | **References** |
| --- | --- | --- | --- | --- | --- |
|  |  |  | **Carriers** | **Patients** |  |
| Ex1 | NG_012247.2:  g.96116980_96138689del  (c.-3251_c.15 +18443del21709bp) | - |  |  | [102] |
| Ex2_3 | c.16_212dup | Unclear | ~1/35000 (C) | **~1/750 (C)** | [82,93,103] |
| Ex3 | c.172_173del | V58Gfs81X |  |  | [18] |
| IVS2_3 | c.70_212del | Y24fs34X |  |  | [98] |
| Ex4 | c.265del | D89fs94X | **~1/29000 (C)** | **~1/515 (C)** | [12,82,93] |
| IVS4 | c.329_468del (IVS4ins6kb) | E110fs127X | ~1/6300 (C) | **~1/140 (C)** | [82,93] |
| Ex6 | c.478del | L160fs196X |  |  | [104] |
| Ex6 | c.495del | Q165fs195X | ~1/70000 (C) | **~1/1550 (C)** | [82] |
| IVS7 | c.755−1G>C | 252fs269X | ~1/70000 (C) | **~1/1550 (C)** | [82] |
| Ex8 | c.845delG | D283fsX285 | ~1/70000 (C) | **~1/1550 (C)** | [82] |
| Ex9 | c.852_855del c.851_854del | M285fs287X  R284fs286X | **~1/110 (C)** | **~1/2 (C)** | [10,12,66-68,78, 82,93,99] |
| Ex9 | c.933_934insGCAG | A312fsX317 | ~1/70000 (C) | **~1/1550 (C)** | [82] |
| Ex10 | c.985_986insT | A329fsX372 | ~1/70000 (C) | **~1/1550 (C)** | [78] |
| Ex16 | c.1706_1707del | S331fsX363 | ~1/70000 (C) | **~1/1550 (C)** | [82] |
| Ex11 | c.1092_1095del | F365fs407X | **~1/3700 (C)** | **~1/120 (C)** | [78,82,93,95] |
| Ex11 | c.1146del | R383fs407X |  | **~1/1500 (J)** | [10] |
| Ex12 | c.1192_1193del | L398fs407X |  |  | [105] |
| Ex14 | c.1375del | A459fs507X |  |  | [10] |
| IVS14_15 | c.1453_1591dup | M532fs560X |  |  | [15] |
| Ex16 | c.1610_1612delinsAT | L537fs538X |  |  | [14] |
| Ex16 | c.1638_1660dup | A554fs570X | **~1/670 (C)** | **~1/13 (C)**  **~1/35 (J)** | [10,12,18,66,68, 82,93,95,99] |
| Ex16_IVS17 | NC_000007.14:  g.96121409_96121924del  (Ex16+74_IVS17-32del516;c.1666_1842-31del) | Q556fs565X |  | **~1/110 (J)** | [10,106] |
| IVS16 | c.1750_1751[ins  NM_138459.3:2672_24;1750+72_1751-4dup]  (IVS16ins3kb) | A584fs585X | ~1/300 (C) | **~1/7 (C)** | [10,12,67,68,78, 82,93,107] |
| Ex17 | c.1799dup | Y600X |  | **~1/75 (J)** | [10,99] |

***Table 3.*** *List of known nonsense mutations involved in citrin deficiency with their frequencies.* *Markings are as in Table 1.*

| **Exon** | **Nonsense mutation** | **Amino acids** | **Frequency** | | **References** |
| --- | --- | --- | --- | --- | --- |
|  |  |  | **Carriers** | **Patients** |  |
| Ex2 | c.46G>T | E16X |  | **~1/750 (J)** | [10] |
| Ex3 | c.72T>A | Y24X | ~1/35000 (C) | **~1/750 (C)** | [82,93] |
| Ex3 | c.127C>T | R43X |  |  | [18] |
| Ex5 | c.448G>T | E150X |  |  | [93] |
| Ex6 | c.475C>T | Q159X | ~1/35000 (C) | **~1/750 (C)** | [82,83,93] |
| Ex6 | c.493C>T | Q165X | ~1/70000 (C) | **~1/1550 (C)** | [82] |
| Ex6 | c.550C>T | R184X | **~1/6000 (C)** | **~1/200 (C)** | [12,66,78,82,95] |
| Ex7 | c.674C>A | S225X | **~1/550 (J)** | **~1/15 (J)** | [10,68,99,108] |
| Ex8 | c.775C>T | Q259X | **~1/2500 (C)** | **~1/100 (J)** | [12,78,82,93] |
| Ex8 | c.847G>T | G283X |  | **~1/750 (C)** | [82,93] |
| Ex10 | c.955C>T | R319X | **~1/3700 (C)** | **~1/100 (C)** | [12,78,82,93,97] |
| Ex11 | c.1063C>T | R355X |  |  | [18,82] |
| Ex11 | c.1078C>T | R360X | ~1/13500 (C) | **~1/300 (C)**  **~1/150 (J)** | [10,78,82,93,108] |
| Ex12 | c.1089C>T | Q397X |  | **~1/100 (C)** | [10] |
| Ex14 | c.1381G>T | E461X | ~1/70000 (C) | **~1/1550 (C)** | [82] |
| Ex14 | c.1399C>T | R467X | **~1/2100 (C)** | **~1/60 (C)** | [12,82,93,105,109] |
| Ex16 | c.1645C>T | Q549X |  | **~1/50 (K)**  **~1/300 (J)** | [68,93,108] |
| Ex16 | c.1736G>A | W579X |  |  | [93] |
| Ex17 | c.1801G>T | R605X |  | **~1/85 (J)** | [10,68,99] |
| Ex17 | c.1813C>T | E601X |  | **~1/100 (C)**  **~1/50 (J)** | [10,108] |

***Table 4.*** *List of known missense mutations involved in citrin deficiency with their frequencies. Markings are as in Table 1.*

| **Exon** | **Missense mutation** | **Amino acids** | **Frequency** | | **References** |
| --- | --- | --- | --- | --- | --- |
|  |  |  | **Carriers** | **Patients** |  |
| E1 | c.2T>C | M1_F34del |  |  | [67,103] |
| Ex3 | c.74C>A | A25E |  |  | [18] |
| Ex3 | c.103A>G | M35V | ~1/70000 (C) | **~1/1550 (C)** | [82] |
| Ex3 | c.115G>T | D39T |  |  | [110] |
| Ex4 | c.221C>T | S74F |  | **~1/70 (K)** | [68] |
| Ex4 | c.254T>C | L85P | ~1/70000 (C) | **~1/1550 (C)** | [78,95] |
| Ex4 | c.284C>A | A95D |  |  | [92] |
| Ex4 | c.287T>C | F96S | ~1/70000 (C) | **~1/1550 (C)** | [78] |
| Ex5 | c.415G>A | G139R |  |  | [93] |
| Ex5 | c.443A>G | Y148C |  |  | [93] |
| Ex6 | c.527G>T | G176V |  |  | [93] |
| Ex7 | c.754G>A | E252K | ~1/9800 (C) | **~1/220 (C)** | [82] |
| Ex8 | c.790G>A | V264I | ~1/70000 (C) | **~1/1550 (C)** | [82,111] |
| Ex8 | c.848G>T | G283V |  |  | [19] |
| Ex10 | c.998G>A | G333D | ~1/34000 (C) | **~1/750 (C)** | [82,93,112] |
| Ex11 | c.1048G>A | D350N | ~1/14000 (C) | **~1/300 (C)** | [82,93] |
| Ex11 | c.1063C>G | R355G | **~1/29500 (C)** | **~1/400 (C)** | [82,93] |
| Ex11 | c.1064G>A | R355Q | ~1/8500 (C) | **~1/190 (C)** | [12] |
| Ex11 | c.1157G>T | G386V |  |  | [105] |
| Ex11 | c.1177G>A | G393S | ~1/10000 (C) | **~1/220 (C)** | [68] |
| Ex12 | c.1215G>T | K405N | ~1/34000 (C) | **~1/770 (C)** | [82,93] |
| Ex13 | c.1231G>A | V411M | ~1/3100 (C) | **~1/70 (C)** | [12,82,93] |
| Ex13 | c.1307-1308delinsAA | G436E |  |  | [13] |
| Ex14 | c.1336A>C | T446P |  |  | [10] |
| Ex14 | c.1349A>G | E450G | ~1/70000 (C) | **~1/1550 (C)** | [78] |
| Ex14 | c.1354G>A | V452L |  |  | [113] |
| Ex14 | c.1358A>G | K453R |  |  | [105] |
| Ex14 | c.1364G>T | R455L | ~1/23000 (C) | **~1/500 (C)** | [82,93] |
| Ex15 | c.1420G>T | V474M |  |  | [114] |
| Ex15 | c.1465C>T | C489R |  |  | [14] |
| Ex15 | c.1478A>G | D493G |  | **~1/300 (J)** | [108,115] |
| Ex15 | c.1498T>G | Y500D |  |  | [93] |
| Ex 15 | c.1511A>G | Y504C |  | **~1/300(J)** | [108] |
| Ex16 | c.1592G>A | G531D |  | **~1/85 (J)** | [10,68,116] |
| Ex16 | c.1622C>A | A541D | ~1/3400 (C) | **~1/75 (C)** | [12,82,93,97] |
| Ex16 | c.1637C>G | T546R |  |  | [10,18,116] |
| Ex16 | c.1637C>T | T546M |  | **~1/650 (J)** | [10] |
| Ex16 | c.1658G>A | R553Q |  |  | [83] |
| Ex17 | c.1754G>A | R585H | ~1/70000 (C) | **~1/1550 (C)** | [78,95] |
| Ex17 | c.1763G>A | R588Q |  |  | [10,100] |
| Ex17 | c.1763G>C | R588P |  |  | [68] |
| Ex17 | c.1781G>A | S589F |  |  | [19] |
| Ex17 | c.1775A>C | Q592P | ~1/34000 (C) | **~1/750 (C)** | [82,93] |
| Ex17 | c. 1781G>A | G594D |  |  | [19,29,30] |
| Ex17 | c.1793T>G | L598R |  | **~1/300 (J)** | [108,116] |
| Ex17 | c.1801G>A | E601K | ~1/34000 (C) | **~1/750 (C)** | [10,78,95] |

**Literature**

92. Takahashi, H. *et al.* (2006) A case of adult-onset type II citrullinemia--deterioration of clinical course after infusion of hyperosmotic and high sugar solutions. *Med Sci Monit* 12, CS13-15

93. Song, Y.Z. *et al.* (2013) SLC25A13 gene analysis in citrin deficiency: sixteen novel mutations in East Asian patients, and the mutation distribution in a large pediatric cohort in China. *PLoS One* 8, e74544

94. Lin, W.X. *et al.* (2021) Neonatal Intrahepatic Cholestasis caused by Citrin Deficiency: *In vivo* and *in vitro* studies of the aberrant transcription arising from two novel splice-site variants in SLC25A13. *Eur J Med Genet* 64, 104145

95. Fu, H.Y. *et al.* (2011) The mutation spectrum of the SLC25A13 gene in Chinese infants with intrahepatic cholestasis and aminoacidemia. *J Gastroenterol* 46, 510-518

96. Lin, W.-X. *et al.* (2012) Multiple ovarian antral follicles in a preterm infant with neonatal intrahepatic cholestasis caused by citrin deficiency: A clinical, genetic and transcriptional analysis. *Gene* 505, 269-275

97. Song, Y.Z. *et al.* (2008) [Identification and diagnosis of three novel mutations in SLC25A13 gene of neonatal intrahepatic cholestasis caused by citrin deficiency]. *Chin J Pediatr* 46, 411-415

98. Wong, L.-J.C. *et al.* (2008) Utility of Oligonucleotide Array–Based Comparative Genomic Hybridization for Detection of Target Gene Deletions. *Clin. Chem.* 54, 1141-1148

99. Yasuda, T. *et al.* (2000) Identification of two novel mutations in the SLC25A13 gene and detection of seven mutations in 102 patients with adult-onset type II citrullinemia. *Hum Genet* 107, 537-545

100. Nakamura, M. *et al.* (2011) The Characteristics of Food Intake in Patients with Type II Citrullinemia. *J Nutr Sci Vitaminol* 57, 239-245

101. Zhang, L. *et al.* (2019) Identification of a novel splicing mutation in the SLC25A13 gene from a patient with NICCD: a case report. *BMC Pediatr* 19, 348

102. Zhang, Z.H. *et al.* (2017) Molecular diagnosis of citrin deficiency in an infant with intrahepatic cholestasis: identification of a 21.7kb gross deletion that completely silences the transcriptional and translational expression of the affected SLC25A13 allele. *Oncotarget* 8, 87182-87193

103. Zhang, Z.H. *et al.* (2012) Molecular analysis of SLC25A13 gene in human peripheral blood lymphocytes: Marked transcript diversity, and the feasibility of cDNA cloning as a diagnostic tool for citrin deficiency. *Gene* 511, 227-234

104. Kose, M.D. *et al.* (2020) Clinical findings in five Turkish patients with citrin deficiency and identification of a novel mutation on SLC25A13. *J Pediatr Endocrinol Metab* 33, 157-163

105. Xing, Y.Z. *et al.* (2010) [Studies on the clinical manifestation and SLC25A13 gene mutation of Chinese patients with neonatal intrahepatic cholestasis caused by citrin deficiency]. *Chin J Med Genet*. 27, 180-185

106. Takaya, J. *et al.* (2005) Variant clinical courses of 2 patients with neonatal intrahepatic cholestasis who have a novel mutation of SLC25A13. *Metabolism* 54, 1615-1619.

107. Kim, Y. *et al.* (2016) Malfunction in Mitochondrial β-Oxidation Contributes to Lipid Accumulation in Hepatocyte-Like Cells Derived from Citrin Deficiency-Induced Pluripotent Stem Cells. *Stem Cells Dev* 25, 636-647.

108. Kido, J. *et al.* (2022) Clinical manifestation and long-term outcome of citrin deficiency: report from a nationwide study in Japan. *J Inherit Metab Dis 45, 431-444*

109. Oh, S.H. *et al.* (2017) Biochemical and molecular characteristics of citrin deficiency in Korean children. *J Hum Genet* 62, 305-307

110. Bai, X.L. *et al.* (2017) Clinical features of children with neonatal intrahepatic cholestasis caused by citrin deficiency and analysis on SLC25A13 gene (in Chinese). *Maternal and Child Health Care of China* 32, 4748-4751

111. Zeng, H.S. *et al.* (2014) Inspissated bile syndrome in an infant with citrin deficiency and congenital anomalies of the biliary tract and esophagus: identification and pathogenicity analysis of a novel SLC25A13 mutation with incomplete penetrance. *Int J Mol Med* 34, 1241-1248

112. Song, Y.Z. *et al.* (2009) Neonatal intrahepatic cholestasis caused by citrin deficiency: Clinical and laboratory investigation of 13 subjects in mainland of China. *Dig Liver Dis* 41, 683-689

113. Seker-Yilmaz, B. *et al.* (2017) p.Val452Ile mutation of the SLC25A13 gene in a Turkish patient with citrin deficiency. *Turk J Pediatr* 59, 311-314

114. Zhang, Z.-H. *et al.* (2014) Screening for Five Prevalent Mutations of *SLC25A13* Gene in Guangdong, China: A Molecular Epidemiologic Survey of Citrin Deficiency. *Tohoku J Exp Med* 233, 275-281.

115. Takahashi, Y. *et al.* (2012) An Elderly Japanese Patient with Adult-onset Type II Citrullinemia with a Novel D493G Mutation in the *SLC25A13* Gene. *Intern Med* 51, 2131-2134

116. Wongkittichote, P. *et al.* (2013) Prediction of the functional effect of novel SLC25A13 variants using a *S. cerevisiae model* of AGC2 deficiency. J Inherit Metab Dis 36, 821-830
